# Monitoring a Mandatory Nonmedical Switching Policy from Originator to Biosimilar Infliximab in Patients with Inflammatory Bowel Diseases: A Population-Based Cohort Study

**DOI:** 10.1155/2023/2794220

**Published:** 2023-03-01

**Authors:** Anat Fisher, Jason D. Kim, Colin R. Dormuth

**Affiliations:** ^1^Department of Anesthesiology, Pharmacology and Therapeutics, University of British Columbia, 2176 Health Sciences Mall, Vancouver, BC V6T 1Z3, Canada; ^2^Department of Anesthesiology, Pharmacology and Therapeutics, University of British Columbia Victoria Office, Suite 210, 1110 Government Street, Victoria, BC V8W 1Y2, Canada

## Abstract

**Background:**

On September 5, 2019, British Columbia announced a new policy (the Biosimilars Initiative) to switch from originator to biosimilar infliximab for patients with inflammatory bowel diseases.

**Objective:**

To monitor the impacts of the policy on the use of medications and health services during the first year of the policy.

**Methods:**

In this population-based cohort study, we used administrative health data to construct three historical cohorts and one policy cohort of patients with inflammatory bowel diseases who used the originator infliximab. We then monitored the cumulative incidence of medications and health services. Log-likelihood ratios were used to quantify differences between the policy cohort and the average of the historical cohorts.

**Results:**

The cohorts included 1839–2368 users of the originator infliximab, ages 4–90 years, mean age 43 years. During the first year of follow-up, we found: (1) a 0.9% increase in the first dispensation of infliximab, biosimilar, or originator; (2) a 16.2% increase in infliximab dose escalation; (3) a decrease of 2.4% in the dispensation of antibiotics and a 2.6% decrease in new use of prednison; (4) an anticipated increase in visits to physicians and gastroenterologists to manage switching to biosimilars (24.0%); (5) a 4.0% decrease in discharges from hospital; and (6) a 2.9% decrease in emergency admissions to hospital.

**Conclusion:**

British Columbia's Biosimilars Initiative for nonmedical switching from originator to biosimilar infliximab for inflammatory bowel diseases was not associated with harmful impacts on medications and health services use. An increase in dose escalation was accompanied by an improvement in health status proxies.

## 1. Introduction

Biologic medications have revolutionized the management of Crohn's disease and ulcerative colitis since their introduction in the late 1990s, and hospitalizations and surgeries for patients with inflammatory bowel diseases (IBDs) have decreased [1–6]. Biologic medications have become the “cost driver” of healthcare expenses: overall spending on medications currently accounts for 44–76% of patients' annual healthcare expenses [7, 8]. To optimize healthcare expenses in patients with IBDs, some regulators and providers have advocated for a nonmedical switch from originator biologic medications to lower-priced biosimilar products, on the rationale that savings achieved by switching [9–14] could benefit patients by providing access to additional services or medications, reducing out-of-pocket medication expenses, and, in the case of private insurers, reducing premiums [11].

Some patients with IBDs and physicians have expressed concerns about nonmedical switching to biosimilars [15–17]. The available literature on switching in patients with IBDs was categorized as “weak evidence” using the GRADE approach for rating the certainty of evidence [18, 19] and there have been calls for direct evidence of clinical benefits and harms based on interventional studies, post-marketing observational research, and surveillance of policies on nonmedical switching [16, 17, 18]. Recent interventional and observational studies and systematic literature reviews found similar benefits and harms related to the biosimilar infliximab compared with the originator in adult [20–29, 30] and pediatric patients [31] with IBD. However, data on the impact of policies for a nonmedical switch are lacking [9, 32–34], and in patients with IBDs, the surveillance to date has been limited to switching rates [32].

We monitored the impact of the Biosimilars Initiative for the treatment of IBDs, announced on September 5, 2019 by British Columbia (BC) Ministry of Health. The Initiative targeted patients with IBDs treated with the originator infliximab [35]. The Biosimilars Initiative, a mandatory nonmedical switching policy, provided a financial incentive to encourage patients to switch to a biosimilar product. To maintain infliximab coverage under the provincial drug plan PharmaCare, patients with IBDs who used the originator infliximab were required to switch to the biosimilar infliximab by the end of the first 6 months of the policy (i.e., March 5, 2020). Patients could apply for exceptional approval to continue coverage of the originator product for medical reasons [36]. We aimed to monitor early signals of unintended and harmful impacts of the policy on the use of medication dispensations and health services in patients with IBDs treated with infliximab. Endpoints used for early signal detection were provided by proxy measures for unstable disease, such as an increase in hospitalization or use of pulse steroids and an increase in switching away from infliximab.

## 2. Methods

### 2.1. Study Design and Data Source

The study involved: (1) describing longitudinal trends in the use of biosimilar infliximab among patients with IBDs in BC, Canada and (2) conducting a prospective cohort study to monitor the impact of the new policy on health services use. We used anonymized, linkable administrative data collected by the BC Ministry of Health (PharmaNet, client registry, and Medical Services Plan payment information) and the Canadian Institute for Health Information (National Ambulatory Care Reporting System and Discharge Abstract Database) (Supplementary Table 1). The data comprised prescription medication records from community pharmacies, characteristics of individuals enrolled in the provincial health plan, outpatient visits to physicians or emergency departments, and inpatient hospitalizations.

### 2.2. Use of Biosimilar Infliximab in Inflammatory Bowel Disease Patients

We extracted all infliximab pharmacy records for patients with IBDs who were enrolled in the provincial health plan between August 1, 2018 and August 31, 2020. We identified patients with IBDs based on an infliximab prescription from a gastroenterologist, frequent visits to a gastroenterologist, or a diagnosis of IBD during inpatient hospitalization or visits to physicians and emergency departments. The monthly use of biosimilar infliximab was presented as the percentage of all infliximab prescriptions (originator and biosimilar) that were for the biosimilars. Data on all BC prescriptions and prescriptions accepted by PharmaCare are reported separately for adult and pediatric patients with IBDs.

### 2.3. Rapid Monitoring Cohorts

We established a source population of individuals who were enrolled in the provincial health plan for at least 1 day between March 7, 2016 and September 5, 2019. Beneficiaries of the First Nations Health Authority and federal programs were not included because we did not have access to their data. From the source population, we assembled four cohorts of users of the originator infliximab who had IBD and PharmaCare coverage. The policy cohort comprised individuals with a prescription for originator infliximab during a 6-month identification period between March 7, 2019 and September 4, 2019, the day before policy introduction. The three historical cohorts consisted of individuals with a prescription for the originator infliximab during 6-month identification periods between March 7 and September 4, 2016, 2017, and 2018. We excluded patients not targeted by the policy: those who no longer used the originator infliximab on September 5 following the identification period (i.e., discontinued infliximab, switched to biosimilar infliximab, or switched to a different biologic medication), and those who lacked PharmaCare coverage for infliximab (including those without enrollment in the provincial health plan). Supplementary Table 2 and Supplementary Figure 1 present the cohort inclusion and exclusion criteria and ascertainment windows. Patients were eligible to be included in more than one cohort if they met the inclusion criteria in multiple years. Each cohort was followed from September 5 for 365 days.

### 2.4. Endpoints

Medication endpoints were the dispensation of infliximab, including initial and subsequent dispensations, infliximab dose escalation (defined as a 25% or greater increase in daily dose for the current prescription compared with the average of earlier dispensations), the dispensation of a different biologic medication, the dispensation of antibiotic medication used for IBD-related conditions, and new use of prednisone, defined as the absence of days supply of any systemic corticosteroid in the 6 months prior to the dispensing of prednisone. Supplementary Table 3 lists the medications monitored. For the policy cohort only, we also measured switching to biosimilar infliximab separately for adult and pediatric patients with IBDs. Health services endpoints were outpatient visits to a physician (any specialty) or nurse practitioner, outpatient visits to a gastroenterologist, visits to an emergency department, discharges from a hospital, and emergency admissions to a hospital.

### 2.5. Statistical Analysis

We measured the daily cumulative incidence of medication endpoints and health services use. Cumulative incidence was the percentage of patients who experienced the outcome before and on each day of follow-up. The daily cumulative incidence difference compared the cumulative incidences for the policy cohort to the average cumulative incidence of the historical cohorts (i.e., expected trends in the absence of policy impact). A cumulative incidence difference of zero was interpreted as the absence of a policy impact on an outcome. Likelihood ratios were used to compare the observed cumulative incidence difference to no difference. We defined a signal of an impact of the policy as daily likelihood ratios that were sustained for at least 30 days above a predefined 7.1 threshold (e^1.96^), meaning the observed difference was 7.1 times more likely than a difference of zero. Although the threshold was arbitrary and intended to be an approximate analogue to the alpha of 0.05, we used likelihood ratios rather than statistical testing because the interpretation of a likelihood ratio remains the same regardless of how many times the data are updated [37, 38]. The increase in cumulative incidence of a specific outcome could be transient, i.e., not sustained to the end of the 1-year follow-up. We interpreted a transient increase as patients experiencing the outcome earlier without an overall change in the number of patients experiencing the outcome. The study method has been previously published [33, 34, 38, 39]. Patients with Crohn's disease and ulcerative colitis were not analysed separately, because this was a signal detection analysis, with the goal of conducting quick and efficient analysis. Furthermore, differentiating between the diseases using administrative data is challenging and most validation studies for disease algorithms using administrative data did not differentiate between Crohn's disease and ulcerative colitis.

## 3. Results

### 3.1. The Use of Biosimilar Infliximab in Inflammatory Bowel Diseases

**Figure 1:**
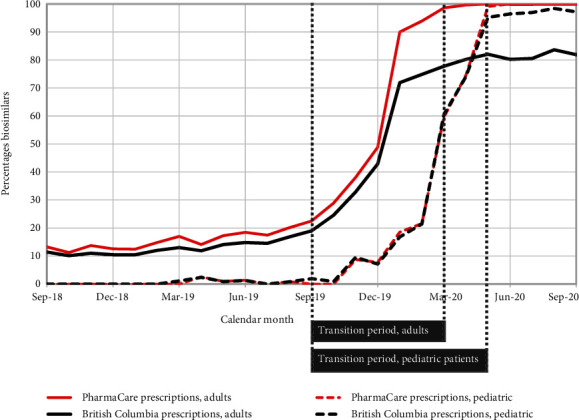
Use of biosimilar infliximab: percentage of all infliximab prescriptions that were for a biosimilar, by month and coverage, for adult and pediatric patients with inflammatory bowel diseases. The transition period of the Biosimilars Initiative started on 5 September 2019 and ended on 4 March 2020 for adults and on 15 May 2020 for pediatric patients. The black lines represent data for all prescriptions in British Columbia; the red lines represent data for prescriptions accepted by PharmaCare, the provincial drug plan.

Between August 2018 and August 2019, before the new BC policy was announced, less than one-fifth of all BC infliximab prescriptions each month were for biosimilars (brand names: Inflectra, Renflexis): 8.1–16.9% of prescriptions for adult patients with IBDs and 0.0%–2.4% for pediatric patients with IBDs (Figure 1). Following the policy launch, biosimilar infliximab comprised 83.7% of all infliximab prescriptions for adults and 98.4% for pediatric patients in August 2020.

### 3.2. Rapid Monitoring of Medication and Health Services Use

The policy cohort consisted of 1839 patients with IBDs who used the originator infliximab during the 6-month identification period (Table 1); the historical cohorts consisted of 1994–2368 patients with IBDs. The most common criterion for excluding patients was the absence of PharmaCare coverage for the originator infliximab (3.5–21.8% of the patients in each cohort). The median age of the cohorts ranged from 41 to 42 years, 53.4–55.0% of patients were male, and Crohn's disease was the most common diagnosis (Table 2).

**Figure 2:**
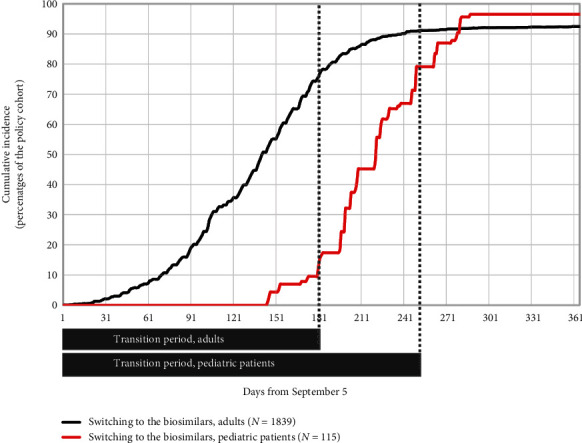
Transition to biosimilar infliximab: percentage of adult and pediatric patients with inflammatory bowel diseases who were previously treated with originator infliximab (brand name Remicade) and switched to biosimilar infliximab following the policy launch. The transition period of the Biosimilars Initiative started on 5 September 2019 and ended on 4 March 2020 for adults and on 15 May 2020 for pediatric patients. Patients with inflammatory bowel diseases were identified based on the algorithm presented in Supplementary Table 1 (policy cohort).

**Figure 3:**
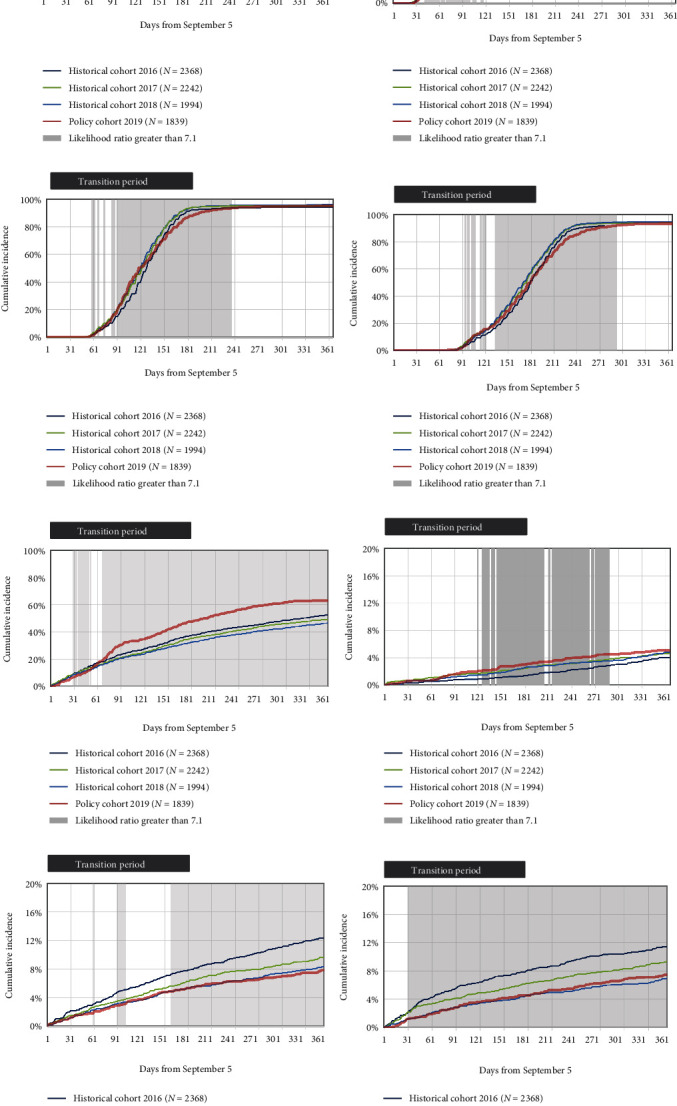
Cumulative incidence of medication outcomes, comparing the policy cohort and the three historical cohorts, for patients of all ages who were treated with infliximab for inflammatory bowel diseases. The medication outcomes are: (a) first dispensation of infliximab (originator or biosimilar) during the 1-year follow-up; (b) second dispensation of infliximab; (c) third dispensation of infliximab; (d) fourth dispensation of infliximab; (e) first event of infliximab (originator or biosimilar) dose escalation, defined as a 25% or greater increase in daily dose for the current prescription compared to the average of earlier dispensations; (f) first dispensation event of a different biologic anti-inflammatory medication (switching); (g) first dispensation event of antibiotic medication (metronidazole, ciprofloxacin); (h) new use of prednisone, defined as the absence of days supply of any systemic corticosteroid in the 6 months before the dispensing of prednisone. Daily likelihood ratios compared the cumulative incidence in the policy cohort to the average among the historical cohorts. A signal of an impact of the policy was defined as likelihood ratios greater than 7.1 (shaded areas) that were sustained for at least 30 days. A transient effect was defined as a period of high likelihood ratios followed by a period in which the likelihood ratio was less than 7.1 (e.g., plates [b] and [c]). Transition periods are presented for adults, who constituted most of the cohorts.

In the year following the policy announcement, 92.3% of the users of originator infliximab in the policy cohort switched to biosimilar infliximab (brand names: Inflectra, Renflexis), and 0.7% of those users switched back to the originator (Figure 2). We used the predefined threshold of a likelihood ratio of 7.1 for 30 days and detected the following changes in the dispensation of infliximab (Figure 3): (1) a sustained 0.9% increase in the cumulative incidence of the dispensation of the first infliximab prescription from day 126 of follow-up onward, (2) a transient 7.5% decrease in dispensation of the third infliximab prescription between days 125 and 232, and (3) a sustained 7.8% decrease in dispensation of the fourth infliximab prescription between days 134 and 292. We observed no signal that the policy impacted the dispensation of the second infliximab prescription. Compared with the average of the historical cohorts, more patients in the policy cohort increased their infliximab dose; the maximal difference in cumulative incidence was +16.2%; and the likelihood ratio exceeded the threshold from day 69 after the policy was implemented. In a *post hoc* analysis, we determined that approximately half (551 of 1136) of the patients with IBDs used the originator infliximab at the time of the first dose increase. A transient increase in switching to a different biologic medication occurred between days 145 and 212 (maximal cumulative incidence difference = +1.1%). We also observed a decrease in the dispensation of antibiotic medications from day 185 of the policy onward (maximal difference = −2.4%), and a decrease in the new use of prednisone throughout the follow-up (maximal difference = −2.6%).

**Figure 4:**
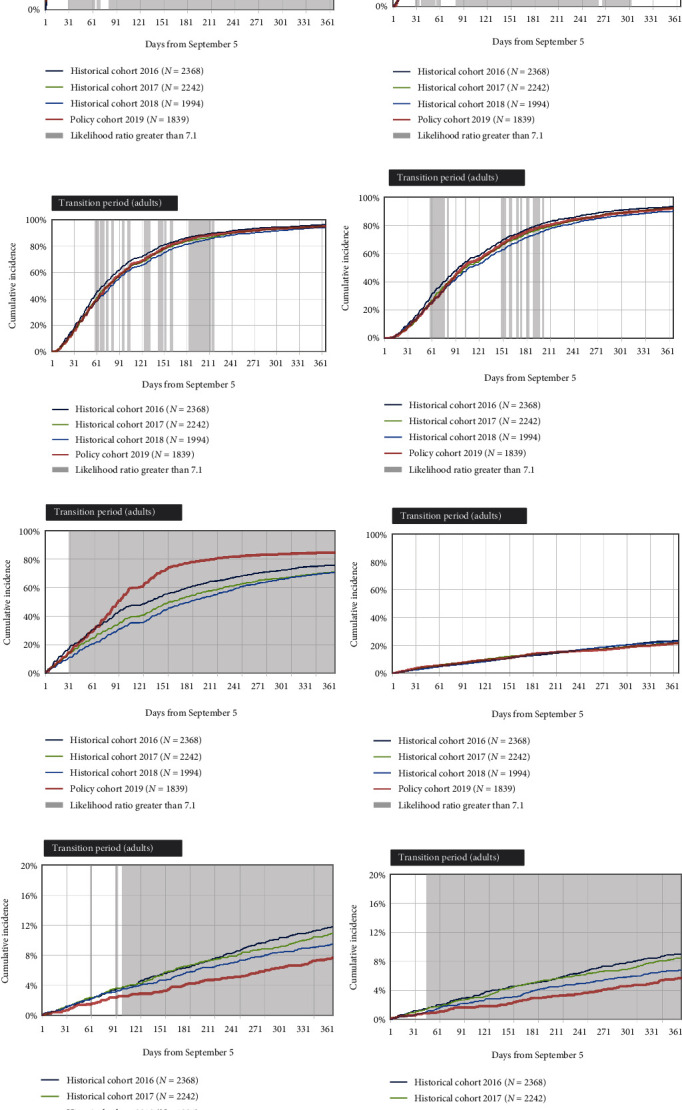
Cumulative incidence of health services outcomes, comparing the policy cohort and the three historical cohorts, for patients of all ages who were treated with infliximab for inflammatory bowel diseases. The health services outcomes are: (a) first visit to a physician or nurse practitioner during the 1-year follow-up; (b) second visit to a physician or nurse practitioner; (c) third visit to a physician or nurse practitioner; (d) fourth visit to a physician or nurse practitioner; (e) first visit to a gastroenterologist; (f) first visit to the emergency department; (g) first discharge from hospital; (h) emergency admission to a hospital. Daily likelihood ratios compared the cumulative incidence in the policy cohort to the average among the historical cohorts. A signal of an impact of the policy was defined as likelihood ratios greater than 7.1 (shaded areas) that were sustained for at least 30 days. A transient effect was defined as a period of high likelihood ratios followed by a period in which the likelihood ratio was less than 7.1 (e.g., plate [b]). Transition periods are presented for adults who constituted most of the cohorts.

Outpatient visits to physicians increased following the implementation of the policy (Figure 4). Likelihood ratios were sustained above the threshold from day 82 onward for the first visit (maximal cumulative incidence difference = +3.5%) and between days 85 and 261 for the second visit (maximal difference = +2.9%). First visit to gastroenterologists increased from day 22 (maximal cumulative incidence difference = +24.0%), discharge from hospital decreased from day 102 onward (maximal difference = −4.0%), and emergency admission to a hospital decreased from day 88 onward (maximal difference = −2.9%). We observed no signal of impact on the first visit to the emergency department, with likelihood ratios sustained below 3.

## 4. Discussion

The new BC nonmedical switching policy had no harmful impact on medication dispensation and health services use among patients with IBDs who switched from originator to biosimilar infliximab. We did not observe increases in hospitalizations, emergency department visits, antibiotics, or prednisone use—all proxy indicators of disease activity. Policy compliance was high: approximately 92% of BC patients with IBDs switched from originator to biosimilar infliximab by the end of the first year of the policy. Most patients tolerated the treatment well: only a few (0.7%) switched back to the originator infliximab, and there was no increase in the rate of switching to a different biologic medication. More patients with IBDs increased their infliximab dose following the policy compared with the average of the historical cohorts. There was an anticipated increase in outpatient visits to physicians, especially gastroenterologists.

While we observed an increase in infliximab dose escalation following the policy launch, previous studies reported conflicting results from switching to a biosimilar: some reported an increase [40, 41], some reported no change [42, 43], and one reported a decrease [44]. Our dose escalation result was unexpected considering plasma medication level monitoring: infliximab plasma levels were similar or higher after switching compared with pre-switch levels [30, 45–47]. The increase in infliximab dose escalation could have resulted from outpatient visits that preceded the switch, which provided “an opportunity for optimization” of infliximab therapy [48, 49]. Alternatively, the increase in dose escalation following the policy launch may be unrelated to the policy or the switch because the increase in the cumulative incidence of dose escalation started early (from month 3, before most users of the originator switched to the biosimilar), and approximately half of dose escalation events occurred while the patients used the originator infliximab (i.e., before the switch). The infliximab dose escalation may be the result of changes in clinical practice and new guideline recommendations for optimizing infliximab treatment from 2019 [50, 51]. The decrease in the use of hospitals, emergency departments, antibiotic medications, and prednisone use suggests there was better disease control following the new guideline recommendations.

Previous observational studies reported patients with IBDs who switched from originator to the biosimilar infliximab experienced better [52] or similar [27–30] clinical responses, including harms, compared with those who continued the originator infliximab. These studies may have been prone to selection or channeling bias because switching was not mandatory [52, 53]. Channeling would have occurred if patients who switched had been in sustained remission or had a milder disease than patients who continued on the originator medication.

Previous observational studies found similar or decreased use of most health services after switching to infliximab biosimilar. The frequencies of visits to emergency departments and hospitalizations in switchers to biosimilar infliximab were comparable to or lower than those of continuers of the originator product [40, 41, 52–54]. Concomitant medication use in switchers, including steroids, morphine, and immunosuppressants, was comparable to that of continuers [41, 52, 55]. Our study results were comparable to those of a previous study that found an increased frequency of visits to gastroenterologists—most likely to discuss the switch—in patients with IBDs who switched from originator to biosimilar infliximab [53].

By using province-wide data, we thus far have studied the largest real-life cohort of patients with IBDs who switched to infliximab biosimilar. Our rapid monitoring analyses should have substantially avoided channeling bias because switching was mandatory and not based on IBD disease morbidity. Data from the three historical cohorts confirmed a stable historical trend upon which we compared the post-policy uptake of infliximab biosimilars.

Our results should be interpreted with caution owing to several limitations. Using real-world data without a concurrent control group, we could not differentiate the effect of the policy from the impacts of other concurrent events, such as modifications to clinical guidelines (as discussed above) or the effects of the COVID-19 pandemic. The Government of BC announced a state of emergency on March 17, 2020 due to the COVID-19 pandemic [56], less than 2 weeks after the end of the 6-month transition period of the policy. The pandemic caused changes in health services use: a decrease in visits to physicians and hospitals [57] and a change in the frequency of medication dispensation because of stockpiling (i.e., panic buying) [58, 59] or manufacturing disruption [60]. In addition, pediatric patients with IBDs had a longer transition period compared with adult patients, until May 15, 2020; therefore, the extension could have diluted the policy impacts. Moreover, there were limitations in our medication data: no records of over-the-counter medications or medications used in hospitals. Lastly, the study was not designed to test hypotheses; therefore, we cannot support or reject the null hypothesis of no difference in health services use after the policy launch. Our method efficiently detected signals of impact: we did not adjust for differences in patients' characteristics, nor did we control for multiple observations of the same individual. However, the method required additional analysis and exploration when a signal was detected.

## 5. Conclusions

During the first year of the BC Biosimilars Initiative, we found no signal of unintended or harmful impacts of the mandatory switching policy that targeted patients with IBDs who were using infliximab. Our results support previous findings of minimal or no harmful effects of switching to a biosimilar on patient health.

## Figures and Tables

**Table 1:** Patient flow for the policy cohort and the three historical cohorts.

	Policy cohort	Historical cohorts		
	2019	2016	2017	2018
Inclusion criteria^†^				
Number of users of originator infliximab^‡^	3,179	3,273	3,363	3,328
Number of patients with an inflammatory bowel disease	2,674	2,637	2,780	2,783
Exclusion criteria, *n* (%) of patients excluded^†^				
Low compliance/discontinuers	97 (3.1)	119 (3.6)	125 (3.7)	157 (4.7)
Switchers	24 (0.8)	7 (0.2)	7 (0.2)	9 (0.3)
Lack of enrollment	20 (0.6)	28 (0.9)	28 (0.8)	33 (1.0)
No PharmaCare	694 (21.8)	115 (3.5)	378 (11.2)	590 (17.7)
Patients in the final cohort, *n*	1,839	2,368	2,242	1,994

^†^Criteria are listed in Supplementary Table 2.

^‡^Brand name Remicade.

The policy cohort comprised individuals with a prescription for originator infliximab during a 6-month identification period between March 7, 2019 and September 4, 2019, the day before policy introduction. The three historical cohorts consisted of individuals with a prescription for the originator infliximab during 6-month identification periods between March 7 and September 4, 2016, 2017, and 2018.

**Table 2:** Patient characteristics for the policy cohort and the three historical cohorts.

	Policy cohort	Historical cohorts		
	2019	2016	2017	2018
Age (years)				
Mean (SD)	43.1 (16.9)	42.6 (16.1)	42.4 (16.5)	43.1 (16.5)
Median (range)	42.0 (5.0–90.0)	42.0 (5.0–89.0)	41.0 (5.0–88.0)	42.0 (4.0–89.0)
Sex, *n* (%)				
Female	827 (45.0)	1104 (46.6)	1033 (46.1)	914 (45.8)
Male	1012 (55.0)	1264 (53.4)	1209 (53.9)	1080 (54.2)
Diagnosis of inflammatory bowel disease, *n* (%) †				
Crohn's disease	959 (52.1)	1186 (50.1)	1129 (50.4)	1040 (52.2)
Ulcerative colitis	466 (25.3)	603 (25.5)	561 (25.0)	488 (24.5)
Both	240 (13.1)	415 (17.5)	378 (16.9)	285 (14.3)
Undetermined	174 (9.5)	164 (6.9)	174 (7.8)	181 (9.1)
Patients with procedure in the previous year, *n* (%)				
Endoscopy of the gastrointestinal tract ‡	541 (29.4)	891 (37.6)	757 (33.8)	527 (26.4)
Surgery of the gastrointestinal tract §	151 (8.2)	219 (9.2)	210 (9.4)	139 (7.0)
Years since first infliximab, mean (SD)	6.7 (3.4)	3.9 (3.2)	4.8 (3.2)	5.8 (3.3)
Number of different medications in the previous year				
Any medication, mean (SD) ¶	6.5 (4.4)	7.2 (4.7)	6.8 (4.5)	6.7 (4.5)
Additional biologic medications, median (range) ††	0.0 (0.0–1.0)	0.0 (0.0–2.0)	0.0 (0.0–1.0)	0.0 (0.0–1.0)
Health services use in the previous year: Mean (SD)				
Number of visits to a physician or nurse practitioner	17.9 (16.8)	31.1 (26.0)	23.0 (19.4)	19.7 (17.8)
Number of nights in hospital	0.6 (5.4)	1.3 (6.9)	0.8 (4.0)	0.6 (3.9)

SD: standard deviation.

^†^Algorithm for identifying inflammatory bowel disease is presented in Supplementary Table 2.

^‡^Procedure codes for endoscopy of the gastrointestinal tract are listed in Supplementary Table 4.

^§^Procedure codes for surgery of the gastrointestinal tract are listed in Supplementary Table 5.

^¶^Including infliximab.

^††^Excluding infliximab (biologic medications are listed in Supplementary Table 3).

The policy cohort comprised individuals with a prescription for originator infliximab during a 6-month identification period between March 7, 2019 and September 4, 2019, the day before policy introduction. The three historical cohorts consisted of individuals with a prescription for the originator infliximab during 6-month identification periods between March 7 and September 4, 2016, 2017, and 2018.

## Data Availability

The data used to support the findings of this study were supplied by the British Columbia Ministry of Health under special agreement and so cannot be made freely available. Requests for access to these data should be made to Population Data BC (the requirements for data access are available from https://www.popdata.bc.ca/data_access/DAR_process).

## Supplementary Materials

Supplementary Materials
Supplementary Table 1 British Columbia Ministry of Health databases. Supplementary Table 2 Inclusion and exclusion criteria for the four cohorts. Supplementary Figure 1 A rapid monitoring cohort of users of originator infliximab for inflammatory bowel diseases. Supplementary Table 3 Biologic medications used for inflammatory bowel diseases. Supplementary Table 4 Procedure codes for colonoscopy and endoscopy of the gastrointestinal tract. Supplementary Table 5 Procedure codes for surgery of the gastrointestinal tract.
