# Supplementary material for: Monitoring a Mandatory Nonmedical Switching Policy from Originator to Biosimilar Infliximab in Patients with Inflammatory Bowel Diseases: A Population-Based Cohort Study
Source: Gastroenterol Res Pract. 2023 Mar 1;2023:2794220. doi: 10.1155/2023/2794220 (PMC9995207; doi:10.1155/2023/2794220)
Supplement: Supplementary Materials — Supplementary Table 1 British Columbia Ministry of Health databases. Supplementary Table 2 Inclusion and exclusion criteria for the four cohorts. Supplementary Figure 1 A rapid monitoring cohort of users of originator infliximab for inflammatory bowel diseases. Supplementary Table 3 Biologic medications used for inflammatory bowel diseases. Supplementary Table 4 Procedure codes for colonoscopy and endoscopy of the gastrointestinal tract. Supplementary Table 5 Procedure codes for surgery of the gastrointestinal tract. [file 2794220.f1.docx]

**Monitoring a mandatory nonmedical switching policy from originator to biosimilar infliximab in patients with inflammatory bowel diseases: A population-based cohort study**

Supplementary material

**Supplementary Table 1**. **British Columbia Ministry of Health databases**

Patient level data were linked using anonymized, deidentified, unique patient numbers

| **D*atabase*** | **D*escription of data*** |
| --- | --- |
| PharmaNet | Prescription medication dispensing records from community pharmacies. No information on medication dispensed in hospitals, over the counter medication, and sample provided by physicians. No information on individuals enrolled in the First Nations Health Authority and federal programs. |
| Client registration file | Deidentified listing on the registration and demographics of individuals enrolled in the provincial health plan. The provincial public health plan is available to eligible residents^1^ (Canadian citizens and permanent residents) and covers the cost of medically-necessary insured doctor services. |
| Medical Services Plan (MSP) payment information | MSP insured medical fee-for-service and alternate payment services provided by general practitioners and specialists, as well as MSP insured services provided by other health practitioners. |
| National Ambulatory Care Reporting System (NACRS) | Hospital-based and community-based ambulatory care (day surgery, outpatient and community-based clinics and emergency departments) in participating facilities. |
| Discharge Abstract Database (DAD) | Captures administrative, clinical, and demographic information on hospital discharges and day surgeries. |

1. For eligibility criteria please refer to <https://www2.gov.bc.ca/gov/content/health/health-drug-coverage/msp/bc-residents/eligibility-and-enrolment/are-you-eligible>. (last access May 27, 2022)

**Supplementary Table 2**. Inclusion and exclusion criteria for the four cohorts

| **The criterion** | **Definition** | **Ascertainment window** |
| --- | --- | --- |
| Inclusion criteria | | |
| British Columbia residents | Individuals covered under the provincial Medical Services Plan | March 7, 2016– September 5, 2019 |
| Users of originator infliximab | A prescription for originator infliximab (brand name Remicade) was filled in one of British Columbia’s community pharmacies | Identification period: the 182 days before September 5 in the years 2016, 2017, 2018, and 2019 |
| Inflammatory bowel disease | At least one refill of originator infliximab prescribed by a gastroenterologist, or | During the identification period |
|  | at least two visits to a physician’s office within one year with a diagnosis of Crohn’s disease or ulcerative colitis (ICD-9 codes 555.xx and 556.xx), or | During the five years before the last infliximab in the identification period |
|  | at least one discharge from a hospital or visit to an emergency department with these diagnoses (ICD-10 codes K50.xx and K51.xx), or | During the five years before the last infliximab in the identification period |
|  | at least five visits to a gastroenterologist | During the five years before the last infliximab in the identification period |
| Exclusion criteria | | |
| Low compliance/ discontinuers | Defined as no medication supply on May 27 + no refill in the 84 days before (excluding) May 27. Mean days supplied in originator infliximab prescriptions in the identification period were 43.7 days; median 42 days | From the date of the last originator infliximab prescription in the identification period to May 27 |
| Switchers | A pharmacy record of   1. a different biologic: rituximab (ATC classification code L01XC02); abatacept (L04AA24); tofacitinib (L04AA29); etanercept (L04AB01); adalimumab (L04BA04); certolizumab pegol (L04AB05); golimumab (L04AB06); anakinra (L04AC03); ustekinumab (L04AC05); tocilizumab (L04AC07); secukinumab (L04AC10); brodalumab (L04AC12); ixekizumab (L04AC13); guselkumab (L04AC16) 2. biosimilar infliximab | From the date of the last originator infliximab prescription in the identification period to May 27 |
| Short follow-up | Lack of continuous health plan enrollment | On May 27 and the following 30 days |
| No PharmaCare coverage | Patients for whom PharmaCare did not accept any of the prescriptions for originator infliximab | During the identification period |

**Cohort entry date**

September 5

Day 1

At least one refill of originator infliximab [Days -183, 0]

Inclusion based on inflammatory bowel diseases [Days -1825, 1]

Exclusion of patients with low compliance, or discontinuers or switchers

[Days -183, 1]

Exclusion of patients without continuous enrollment

[Days 1, 31]

Exclusion if no refill of originator infliximab was accepted by PharmaCare [Days -183, 0]

Follow-up [Days 1, 365]

time

**Supplementary Figure 1.** A rapid monitoring cohort of users of originator infliximab for inflammatory bowel diseases.

**Supplementary Table 3.** Biologic medications used for inflammatory bowel diseases

| **Anatomical Therapeutic Chemical (ATC) code** | **Generic name** |
| --- | --- |
| L04AA23 | natalizumab |
| L04AA29 | tofacitinib citrate |
| L04AA33 | vedolizumab |
| L04AB04 | adalimumab |
| L04AB05 | certolizumab pegol |
| L04AB06 | golimumab |
| L04AC05 | ustekinumab |

**Supplementary Table 4**. Procedure codes for colonoscopy and endoscopy of the gastrointestinal tract

| **Procedure code** | **Description** |
| --- | --- |
| Canadian Classification of Health Interventions | |
| 2.NK.70.^^, except 2.NK.70.DA or 2.NK.70.LA | Inspection, small intestine, using endoscopic approach |
| 2.NK.71.^^, except 2.NK.71.DA, 2.NK.71.HA or 2.NK.71.LA | Biopsy, small intestine, using endoscopic approach |
| 2.NM.70.^^ except 2.NM.70.DA or 2.NM.70.LA | Inspection, large intestine, using endoscopic approach |
| 2.NM.71.^^, except 2.NM.71.DA, 2.NM.71.HA or 2.NM.71.LA | Biopsy, large intestine, using endoscopic approach |
| Fee items from the BC Medical Services Commission payment schedule* | |
| 00373 | Colonoscopy with flexible colonoscope – biopsy |
| 00374 | Colonoscopy with flexible colonoscope – polyp removal |
| 00572 | Pediatric colonoscopy with flexible colonoscope – patients 16 years of age and under |
| 07581 | Colonoscopy |
| 07582 | Colonoscopy – biopsy |
| 07583 | Colonoscopy – polyp removal |
| 10231 | Colonoscopy with flexible colonoscope (PHSA program†) |
| 10233 | Colonoscopy with flexible colonoscope – biopsy (PHSA program) |
| 10234 | Colonoscopy with flexible colonoscope – polyp removal (PHSA program) |
| 10730 | Colonoscopy, flexible transabdominal via colostomy |
| 10731 | Colonoscopy, flexible, proximal to splenic flexure; diagnostic with or without collection of specimen(s) by brushing or washing |
| 10732 | Colonoscopy, flexible, proximal to splenic flexure; with removal of foreign body |
| 10733 | Colonoscopy, flexible, proximal to splenic flexure; with control of bleeding, any method |
| 10735 | Rectal endoscopy using ultrasound (radial/linear) |
| 33373 | Colonoscopy with flexible colonoscope – biopsy |
| 33374 | Colonoscopy with flexible colonoscope – polyp removal |
| 70549 | Pediatric esophagogastroduodenoscopy in a patient 16 years of age or under |

* Details are available at <https://www2.gov.bc.ca/gov/content/health/practitioner-professional-resources/msp/physicians/payment-schedules/msc-payment-schedule>.

† The Provincial Health Services Authority (PHSA) program is colorectal cancer screening program. Details are available at <https://archive.news.gov.bc.ca/releases/news_releases_2005-2009/2009hserv0002-000075.htm>.

**Supplementary Table 5.** Procedure codes for surgery of the gastrointestinal tract

| **Procedure code** | **Description** |
| --- | --- |
| Canadian Classification of Health Interventions | |
| 1.NK.^^.^^, excluding 1.NK.27.^^ | Therapeutic interventions on the small intestine |
| 1.NM.^^.^^, excluding 1.NM.27.^^ | Therapeutic interventions on the large intestine |
| 1.NP.^^.^^, excluding 1.NP.35.^^ | Therapeutic interventions on the small and large intestine |
| 1.NQ.^^.^^, excluding 1.NQ.12.^^, 1.NQ.26.^^, or 1.NQ.27.^^ | Therapeutic interventions on the rectum |
| 1.NT.^^.^^,excluding 1.NT.07.^^, 1.NT.26.^^, 1.NT.27.^^, or 1.NT.35.^^ | Therapeutic interventions on the anus |
| Fee items from the BC Medical Services Commission payment schedule | |
| 07452 | Repair of extra-peritoneal rectum, with or without colostomy |
| 07634 | Full thickness repair of iatrogenic intestinal perforation (single) |
| 07635 | Full thickness repair of iatrogenic intestinal perforation (multiple) |
| 07636 | Resection of small intestine – with anastomosis – open |
| 07638 | Rectum and sigmoid anterior resection |
| 07646 | Closure of loop enterostomy, large or small intestine – without resection |
| 07650 | Intestinal obstruction; resection of bands; enterolysis-open |
| 07654 | Intestinal obstruction – plication or insertion of intraluminal tube |
| 07668 | Rectum tumor excision or fulguration – small |
| 07669 | Rectum tumor excision or fulguration – medium |
| 07671 | Anus stricture plastic repair |
| 07673 | Rectum tumor excision or fulguration – large |
| 07681 | Anus fissure or excision anal fissure |
| 07690 | Anoplasty; for imperforate anus |
| 07691 | Anus imperforate – simple incision (operation only) |
| 08170 | Preparation of intestinal segment and reanastomosis |
| 72620 | Resection of small intestine – with enterostomy; without anastomosis (does not include separate enterostomies or resections) – open |
| 72647 | Intestinal strictoplasy (enterotomy and enterorrhaphy) with or without dilation for intestinal obstruction – single |
| 72648 | Intestinal strictoplasy (enterotomy and enterorrhaphy) with or without dilation for intestinal obstruction – multiple (two or more) |
| 72650 | Intestinal obstruction, resection of bands, enterolysis-laparoscopic |
| 72667 | Division of stricture of rectum (includes endoscopy) – operation only |
| 72720 | Resection of small intestine with enterostomy; without anastomosis (does not include separate enterostomies or resections) – laparoscopic |
| 72736 | Resection of small intestine with anastomosis – laparoscopic |

* Details are available at <https://www2.gov.bc.ca/gov/content/health/practitioner-professional-resources/msp/physicians/payment-schedules/msc-payment-schedule>.
